# Molecular Detection and Characterization of the *Staphylococcus epidermidis* and *Staphylococcus haemolyticus* Isolated from Hospitalized Patients and Healthcare Workers in Iran

**DOI:** 10.1155/2023/3775142

**Published:** 2023-01-06

**Authors:** Mohammad Ali Noshak, Mohammad Ahangarzadeh Rezaee, Alka Hasani, Mehdi Mirzaii, Mohammad Yousef Memar, Taher Azimi, Maryam Ebrahimi, Alireza Dolatyar Dehkharghani

**Affiliations:** ^1^School of Medicine, Shahroud University of Medical Sciences, Shahroud, Iran; ^2^Immunology Research Center, Tabriz University of Medical Sciences, Tabriz, Iran; ^3^Infectious and Tropical Diseases Research Center, Tabriz University of Medical Sciences, Tabriz, Iran; ^4^Department of Laboratory Science, Faculty of Paramedicine, Tabriz University of Medical Sciences, Tabriz, Iran; ^5^Clinical Research Development Unit of Children Educational, Research and Treatment Center, Tabriz University of Medical Sciences, Tabriz, Iran; ^6^Department of Medical Microbiology, Faculty of Medicine, Tabriz University of Medical Sciences, Tabriz, Iran; ^7^Department of Bacteriology and Virology, School of Medicine, Shiraz University of Medical Sciences, Shiraz, Iran; ^8^Research Center of Health Reference Laboratories, Ministry of Health and Medical Education, Tehran, Iran

## Abstract

**Background:**

The present study is aimed at surveying the antibiotics resistance profile, biofilm formation ability, staphylococcal cassette chromosome *mec* (SCC*mec*) types, and molecular epidemiology of *Staphylococcus epidermidis* and *Staphylococcus haemolyticus* isolated from hospitalized patients and healthcare workers in four teaching hospitals in Iran.

**Methods:**

In total, 43 *Staphylococcus epidermidis* and 12 *Staphylococcus haemolyticus* were isolated from hospitalized patients, and 19 *Staphylococcus epidermidis* and 7 *Staphylococcus haemolyticus* isolated from healthcare workers were included in the present study. The antimicrobial resistance profile of isolates was determined using the disk diffusion method. Moreover, the resistance of isolates to methicillin was identified using the cefoxitin disk diffusion test. The microtiter-plate test was used for quantifying biofilm formation. Moreover, the frequency of *icaA* and *icaD* genes was determined using PCR assay. The molecular epidemiology of methicillin-resistant isolates was determined using SCC*mec* typing and pulsed-field gel electrophoresis methods.

**Results:**

Among all coagulase-negative staphylococci isolates, the highest resistance rate (81.5%) was seen for cefoxitin and cotrimoxazole. All of the isolates were susceptible to linezolid. Out of the 66 *mecA*-positive isolates, the most common SCC*mec* type was the type I (*n* = 23; 34.8%) followed by type IV (*n* = 13; 19.7%). Using pulsed-field gel electrophoresis (PFGE) assay, 27 PFGE types including 14 common types and 13 singletons were obtained among 51 methicillin-resistant *S. epidermidis* (MRSE) isolates. Moreover, among 12 methicillin-resistant *S. haemolyticus* (MRSH) isolates, 8 PFGE types were detected, of which 5 PFGE types were singletons.

**Conclusion:**

The high rate of resistance to antibiotics as well as the possibility of cross-infection shows the importance of a pattern shift in the management and controlling programs of coagulase-negative staphylococci, especially in healthcare centers. *Clinical trial registration.* The present study is not a clinical trial study. Thus, a registration number is not required.

## 1. Introduction

In recent years, coagulase-negative staphylococci (CoNS) as part of the skin and mucous membranes microbiota of humans had a profound influence on human life and health [1]. Among the CoNS, *Staphylococcus epidermidis* (*S. epidermidis*) and *Staphylococcus haemolyticus* (*S. haemolyticus*) are the most common species and can cause several infections, especially in patients with debilitating underlying diseases. These bacteria become resistant to a variety of antibiotics, especially beta-lactam drugs [2, 3]. It is well known that the ability of biofilm formation makes bacteria to resistant to antimicrobial agents and to the host immune systems. Therefore, biofilm formation capacity is one of the most significant virulence factors that could lead to CoNS pathogenicity [4]. The emergence and increase of methicillin-resistant CoNS isolates are known as a public health concern [5]. In most cases, the *mecA* gene is responsible for the emergence of methicillin resistant (MR) isolates. It is revealed that MR-CoNS are often resistant to non-*β*-lactam antibiotics such as amikacin, ciprofloxacin, and clindamycin [6]. *mecA* gene carried by mobile genetic elements referred to as a staphylococcal cassette chromosome mec (SCC*mec*) [7]. So far, eleven types of SCC*mec* (types I to XI) have been described. However, only eight types (types I to VIII) have been established. All types I to XI have been described in *S. aureus*. To date, eight types of SCC*mec* (types I to VIII) were identified in CoNS isolates [8, 9]. Notably, it has been reported that CoNS isolates can be transmitted between hospitalized patients (HPs) and healthcare workers (HCWs) [10]. Several studies revealed that the colonized HPs and HCWs are the main source of CoNS isolates [11, 12]. However, considering that CoNS are considered a group of opportunistic pathogens, sufficient attention is not paid to the management and control of these bacteria [10, 13, 14]. Pulsed-field gel electrophoresis (PFGE) is an approved molecular typing method that can help in identifying the genetic relatedness between the staphylococcal isolates and their fingerprinting [15] In the present study, we used PFGE method to identify the genetic relatedness between the *S. epidermidis* and *S. haemolyticus* isolated from HPs and HCWs. Moreover, we surveyed the antibiotic resistance profile and biofilm formation capacity and determined the predominant SCC*mec* types of *S. epidermidis* and *S. haemolyticus* isolated from HPs and HCWs in four teaching hospitals in Iran.

## 2. Materials and Methods

### 2.1. Study Design and Bacterial Isolates

The present cross-sectional study was performed from May 2020 to March 2021 in 3 teaching hospitals in Tabriz (northwest Iran) and one teaching hospital in Shahroud (northeast Iran). In the first step, all participants including HPs and HCWs signed an informed consent form, and clinical samples including blood, urine, tracheal tube, and pleural fluid were collected from them. Swab samples were taken with cotton sterile swabs from the hands of 80 healthcare providers (including 60 nurses and 20 physicians). All samples were cultured on blood agar and mannitol salt agar (Merck, Germany) plates and incubated at 37°C for 24 hours. After overnight incubation, Gram staining was performed on all colonies and phenotypic confirmation of CoNS isolates was carried out with catalase, oxidase, DNase, and coagulase tests, and growth in nutrient broth containing 6% sodium chloride (NaCl). The molecular identification of the *Staphylococcus* genus was performed based on the previously published work by Nahaei et al. [16]. Moreover, to final confirmation of *S. epidermidis* and *S. haemolyticus*, polymerase chain reaction (PCR) *assay* targeted the 16 s*-*23 s ribosomal DNA; internal transcribed spacer (ITS) region was applied based on the previously published studies by Cunha et al. [17] and Couto et al. [18].

### 2.2. Antimicrobial Susceptibility Testing

The antibiotic resistance patterns of *S. epidermidis* and *S. haemolyticus* were determined using the disk diffusion method on the Muller-Hinton agar medium (Merck, Germany). Antibiotics used in the present study were erythromycin (15 *μ*g), cefoxitin (30 *μ*g), ciprofloxacin (5 *μ*g), clindamycin (2 *μ*g), gentamicin (5 *μ*g), rifampicin (5 *μ*g), linezolid (30 *μ*g), and trimethoprim-sulfamethoxazole (1.25/23.75 *μ*g). Antibiotic disks were purchased from Mast company (Mast Group Co., UK). The minimum inhibitory concentration (MIC) of vancomycin (Sigma) was determined by the broth microdilution method. All results were interpreted based on the Clinical and Laboratory Standards Institute criteria (CLSI, 2019) [19]. Resistance to at least one antibiotic from three or more classes of antibiotics was defined as multidrug resistance (MDR) [20].

### 2.3. Assessment of Biofilm Formation

A quantitative assessment of biofilm formation was carried out by the microtiter-plate test (MTP) as described by Mohsenzadeh et al. [21]. Briefly, 2-3 colonies of the tested isolates were cultured in 10 mL of Trypticase Soy Broth (TSB) (Merck, Germany) containing 1% glucose (Sigma) and were incubated for 24 h at 37°C (adjusted to 0.5 McFarland standards). After overnight incubation, the suspension was shaken and then diluted at 1 : 100 in TSB containing 1% glucose. In the next step, 200 *μ*L of the diluted solution (bacterial suspension) was added to each well of a microplate. Microplates were covered and incubated at 37 C for 18 h and following the process of incubation, the bacterial suspension was removed and all wells were washed five times with sterile distilled water. Afterward, the plates were stained with 0.1% crystal violet (Merck, Germany). Finally, the plates were washed and dried, and optical density (OD) was read at 570 nm. If the OD of the isolates was ≥0.12, they were classified as biofilm producers. The cut-off value was selected to differentiate between isolates that generated considerable amounts of biofilm and those that did not, taking into account the OD values for each experiment's negative controls. Also, *S. epidermidis* ATCC 12228 and *S. epidermidis* RP62A were used as negative and positive controls, respectively.

### 2.4. Detection of *mecA, icaA*, and *icaD* Genes by PCR

Total genomic DNA extraction was performed with the Cetyltrimethylammonium Bromide (CTAB) procedure as described by Nahaei et al. [16]. For final confirmation of methicillin-resistant *S. epidermidis* (MRSE) and methicillin-resistant *S. haemolyticus* (MRSH), the PCR method targeted the *mecA* gene was used. PCR conditions, the volume of materials, and primer sequence were set based on a previously published study by de Allori et al. [22]. Moreover, PCR analysis for *icaA* and *icaD* genes was performed using specific primers. The volume of materials, PCR conditions, and primer sequences have been described previously by Arciola et al. [23]. The PCR products were electrophoresed on 1.5% agarose gels in TBE buffer (89 mM Tris base, 89 mM boronic acid, 2 mM Na2, EDTA, pH 8.25). The gel was stained using the DNA-safe stain (SinaClon Co., Iran) and was observed under ultraviolet light. A 100 bp DNA ladder was used as a molecular size indicator.

### 2.5. SCC*mec* Typing

To determine SCC*mec* types I to V among *mecA*-positive isolates, SCC*mec* typing was carried out using the multiplex-PCR method. The primer sequences, PCR condition, and volume of material were set based on a study performed by Ghanbari et al. [24]. DNA was amplified with a thermocycler (Eppendorf, Mastercycler Gradient; Eppendorf, Hamburg, Germany).

### 2.6. Pulsed-Field Gel Electrophoresis (PFGE)

Typing MRSE and MRSH was performed using the PFGE method as previously described by Talebi et al. [3]. Briefly, the enzyme digestion of the plugs was carried out with the restriction enzyme *Sma*I (New England Biolabs). A *Salmonella* serotype *Braenderup* strain (H9812) was used as a molecular size marker (kindly provided by the Research Center of Health Reference Laboratories, Tehran, Iran). In addition, DNA separation was carried out by programming two states under the following conditions in a pulsed-field electrophoresis system (CHEF DR-II; Bio-Rad Laboratories, Hercules, CA, USA) at the temperature of 14°C; voltage 6 V/cm; switch angle, 120°C; switch ramp 1-30 second for 20 hours. Ethidium bromide (SinaClon, Iran) 0.5 mg/mL was used for staining gels. For PFGE pattern analysis, BioNumerics software version 7.5 (Applied Maths, St-Martens-Latem, Belgium) was applied. The unweighted pair group method by using mathematical averaging (UPGMA), dice correlation coefficient with 1.5% optimization, and a 1.5% tolerance setting was used for the calculation of dendrograms. Isolates were considered genetically indistinguishable, closely related, possibly related, and different when there were 0, 1, 2, and >3 banding differences, respectively [25].

### 2.7. Statistical Analysis

All results were included in SPSS software version 20 (SPSS Inc., Chicago, IL, USA) and analyzed using the chi-square test. A *P* value of < 0.05 was considered statistically significant.

## 3. Results

### 3.1. Number of Bacteria and Distribution of Samples

In total, 80 clinical samples and 80 swab samples were taken from 80 HPs and 80 HCWs, respectively. The frequency of *S. epidermidis* and *S. haemolyticus* among different clinical samples and in different wards of hospitals are shown in Table 1. Of samples collected from HPs, 55 cultures (68.7%) (43 (53.7%) *S. epidermidis* and 12 (15%) *S. haemolyticus*) were determined to be positive for *S. epidermidis* and *S. haemolyticus*. In contrast, results showed that 19 (23.7%) and 7 (8.7%) swab samples taken from HCWs were positive for *S. epidermidis* and *S. haemolyticus*, respectively. The frequency of *S. epidermidis* and *S. haemolyticus* among clinical samples were as follows: blood (*n* = 36), tracheal tube (*n* = 9), urine (*n* = 5), and pleural fluid (*n* = 5).

In total, the majority of bacteria were recovered from the intensive care unit (ICU) (*n* = 55; 67.9%).

### 3.2. Antimicrobial Susceptibility

The antibiotic resistance profile of *S. epidermidis* and *S. haemolyticus* isolated from HPs and HCWs is shown in Table 2. In total, *S. epidermidis* had a higher rate of resistance to trimethoprim-sulfamethoxazole (83.9%; *n* = 53/62) and cefoxitin (80.6%; *n* = 50/62), respectively*. S. haemolyticus* showed the highest rate of resistance to cefoxitin (80.6%; *n* = 17/19) and ciprofloxacin (78.9%; *n* = 15/19), respectively. Linezolid was the most effective antimicrobial agent against *S. epidermidis* and *S. haemolyticus* isolates. 72.6% (*n* = 45/62) and 78.9% (*n* = 15/19) of *S. epidermidis* and *S. haemolyticus* isolates were MDR, respectively. In total, 80% and 61.5% of CoNS isolated from HPs and HCWs were MDR. The ranges of vancomycin MICs for the HPs isolates and HCWs isolates varied from 0.5-8 *μ*g/mL to 0.5-4 *μ*g/mL, respectively. Moreover, the MIC_50_ and MIC_90_ of vancomycin were 1 *μ*g/mL and 8 *μ*g/mL for HPs and 1 *μ*g/mL and 2 *μ*g/mL for HCWs isolates, respectively.

### 3.3. Biofilm Formation

Results obtained from the MTP assay revealed that 89.1% (*n* = 49/55) of CoNS isolated from HPs had OD ≥ 0.12, indicating that they developed biofilms (Table 3). Out of 49 CoNS isolates with biofilm formation capacity, 24 (43.6%) and 25 (45.4%) were positive and negative for *icaA*/*icaD* genes, respectively. In contrast, out of 6 CoNS isolates without biofilm formation capacity, 2 (3.6%) and 4 (7.3%) were positive and negative to *icaA* and *icaD*, respectively. There was no significant association between biofilm formation and the existence of *icaA*/*icaD* genes (*P* > 0.05). Our finding showed that 80.7% (*n* = 21/26) of CoNS isolates recovered from HCWs have biofilm formation capacity. Moreover, 64.2% of MDR isolates were biofilm producers. The statistical analysis showed a significant relationship between MDR and biofilm formation ability (*P* < 0.05).

### 3.4. Detection of *mecA* Gene and SCC*mec* Types

The distribution of SCC*mec* types is shown in Table 3. Overall, among 62 *S. epidermidi*s and 19 *S. haemolyticus*, 52 (83.9%) and 14 (73.7%) isolates were positive for the *mecA* gene. The SCC*mec* typing method was performed on 66 *mecA*-positive CoNS isolates. Frequency of SCC*mec* types were as follows: SCC*mec* type I (*n* = 23, 34.8%) SCC*mec* type IV (*n* = 13, 19.7%), SCC*mec* type I + III (*n* = 9, 13.6%), SCC*mec* type II (*n* = 4, 6.1%), SCC*mec* type III (*n* = 2; 3%), and SCC*mec* type III + V (*n* = 2; 3%). 19.7% (*n* = 13) of isolates were not typeable.

### 3.5. PFGE

**Figure 1:**
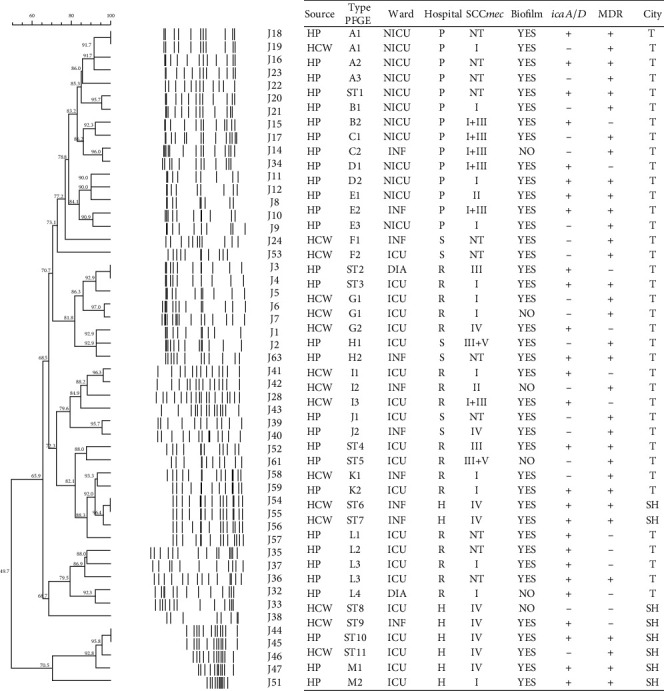
Dendrogram of *Sma*I-PFGE of MRSE isolated from HPs and HCWs. Abbreviations: MRSE: methicillin resistant *S. epidermidis*; ICU: intensive care unit; NICU: neonatal intensive care unit; INF: infectious ward; DIA: dialysis ward; ST: singleton type; NT: not Typeable; T: Tabriz; H: Imam Hossein Hospital; S: Sina Hospital; P: pediatric; R: Imam Reza Hospital.

The clonal diversity of 51 MRSE isolates and 12 MRSH isolates was surveyed using the PFGE method. In total, 2 MRSE and 1 MRSH produced band sizes below 36 kb and were excluded from the study [26]. Out of 51 MRSE isolates, 27 PFGE types including 14 common types (CT: A-N) and 13 singletons were obtained (Figure 1). CT-L was detected in 5 isolates, of which 4 isolates were obtained from HPs and 1 isolate was obtained from HCWs. Results showed that isolates J18 (isolated from the HPs) and J19 (isolated from the HCWs) had completely similar patterns, however, the SCC*mec* types of these isolates were different.

**Figure 2:**
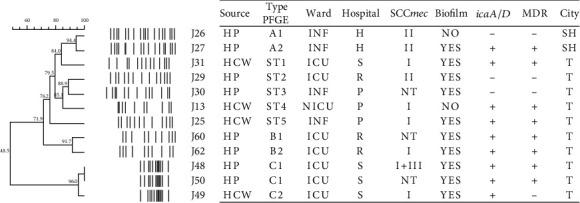
Dendrogram of *Sma*I-PFGE of MRSH isolated from HPs and HCWs. Abbreviations: MRSH: methicillin-resistant *S. haemolyticus*; ICU: intensive care unit; NICU: neonatal intensive care unit; INF: infectious ward; ST: singleton type; NT: not typeable; T: Tabriz; SH: Shahroud Hospital; H: Imam Hossein Hospital; S: Sina Hospital; P: pediatric; R: Imam Reza Hospital.

It was noteworthy that both isolates J18 and J19 had MDR phenotypes. The CT-E consisted of 3 isolates recovered from HCWs and complete genetic similarity was observed between the 2 isolates. As shown in Figure 2, among 12 MRSH isolates, 8 PFGE types were detected, of which 5 PFGE types were singletons. PFGE type C consisted of 2 HPs isolates and 1 HCW isolate.

## 4. Discussion

In general, CoNS isolates are part of the common human microbiota and are considered opportunistic pathogens which can cause several important infections, especially in patients who have medical indwelling equipment [27, 28]. In recent years, CoNS have become resistant to the majority of commonly prescribed antibiotics [13, 24]. In healthcare settings, it is presumed that these pathogens can be transmitted between patients and HCWs. Transmission of these opportunistic pathogens by HCWs could lead to the dissemination of infections in different wards of hospitals [14].

In the present study, we investigated the biofilm formation capacity of *S. epidermidis* and *S. haemolyticus* isolated from HPs and HCWs. Moreover, we surveyed the antibiotic resistance profile and genetic relationship between these CoNS isolates. Results of our study revealed that the resistance rate to several antibiotics including erythromycin, clindamycin, and trimethoprim-sulfamethoxazole was significantly higher among *S. epidermidis* and *S. haemolyticus* isolated from HPs. This may be a result of the indiscriminate use of antibiotics among HPs and the induction of selective pressure. However, there was no significant difference in resistance to gentamicin, ciprofloxacin, rifampicin, and cefoxitin among CoNS isolated from HPs and HCWs.

Our findings showed that 81.8% and 80.8% of CoNS isolated from HPs and HCWs were methicillin-resistant, respectively. Globally, several studies have surveyed the prevalence of resistance to methicillin among CoNS isolates. A study conducted in Iran reported that 74% of the *S. epidermidis* isolates were methicillin-resistant [15]. Another study performed by Cherifi et al. from Belgium reported that 62% and 85% of CoNS isolated from HCWs and patients with catheter-related bloodstream infections were methicillin-resistant, respectively [29]. Liakopoulos et al. from Greece stated that 88.1% of methicillin-resistant CoNS isolates were related to clinical samples collected from chronic hemodialysis patients [30]. Resistances to antibiotics among CoNS isolated from HCWs show an adaptation of the microflora to a pressure induced by the antimicrobial agents' administration in hospital settings, where the colonization may occur.

In the present study, all CoNS isolates were susceptible to linezolid. This finding was in agreement with other studies conducted in Iran [31, 32]. In contrast, a study performed in the USA revealed that resistance to linezolid among CoNS isolates ranged from 1% to 2% [33]. This result may be because linezolid is not prescribed for the treatment of CoNS infections in Iran.

Biofilm formation capacity is known as an important feature used by CoNS isolates for persistence and survival in the host body and hospital settings [4]. Polysaccharide intercellular adhesin (PIA) is encoded by *ica* genes and is the main factor in biofilm formation among CoNS isolates [34]. Our findings revealed that 62.2% and 57.1% of CoNS isolated from HPs and HCWs were positive for *icaA* or *icaD* genes, respectively. In general, the high prevalence rates of *icaA* and *icaD* genes in CoNS isolated from HPs is a serious alarm and suggest that most CoNS isolates recovered from these patients are virulent. In the present study, results obtained from PCR assay revealed that the frequency of the *icaA* and *icaD* genes was more in MRSH compared to MRSE (78.8% versus 55.8%). In line with our study, several studies have reported that *S. epidermidis* and *S. haemolyticus* are potential biofilm producers [35, 36]. Also, it was shown that biofilm formation was associated with the presence of *icaA* and *icaD* genes. In contrast, the results of a study showed that biofilm formation in *S. haemolyticus* is independent of the *ica* genes [37]. However, Pinheiro et al. have reported that *S. haemolyticus* isolates with biofilm-forming ability harbor *ica* genes [28]. Other genes such as *aap*, *bap*, and *bhp* may be involved in *ica*-independent biofilm formation [38].

In the present study, the SCC*mec* types I (34.8%) and IV (19.7%) had the highest frequency among all MRSE and MRSH isolates, respectively. Machado et al. from Brazil reported that SCC*mec* type I was the most prevalent SCC*mec* type among *S. haemolyticus*. In contrast, they showed that SCC*mec* type III was frequently detected among *S. epidermidis* isolates. [39]. In a study performed by Zong et al. in China, the SCC*mec* type III was the most prevalent SCC*mec* type among methicillin-resistant CoNS isolates [40]. Moreover, SCC*mec* type IV has been reported as the most frequent SCC*mec* type among CoNS isolates in Mexico [27] and France [41].

It is revealed that several factors such as differences in geographical locations and host species can be affected the distribution of various types of SCC*mec* in methicillin-resistant CoNS isolates. The use of an appropriate method can be helpful in molecular epidemiology studies. SCC*mec* typing method is not an accurate and reliable procedure for the epidemiological study of CoNS isolates [4], therefore, we used from PFGE method to survey the genetic relatedness between the *S. epidermidis* and *S. haemolyticus* isolated from HPs and HCWs.

PFGE is the most appropriate method for CoNS epidemiology and surveillance analysis [25]. In the present study, PFGE showed high genetic diversity in the CoNS isolated from HPs. High genetic diversity among CoNS isolates revealed that the source of most isolates may be the patients themselves; however, the incidence of cross infections between the patients is also possible. The presence of CoNS isolates with similar PFGE types among HPs and HCWs suggested that HCWs can be a reservoir for transferring CoNS to HPs and vice versa.

In the present study, most of the methicillin-resistant CoNS isolates were isolated from ICU staff and patients. Some of these isolates showed identical patterns which confirmed person-to-person transmission within the ICU ward. However, the isolates from other wards showed a high heterogeneity, which may be due to the transfer of personnel and/or patients among different wards of the hospital. In general, CoNS isolates with similar PFGE types can be grouped into different SCC*mec* types [42, 43]. The diversity of SCC*mec* among PFGE types may result from the high rate of SCC*mec* acquisition and frequent insertion/excision of SCC*mec* in the CoNS chromosome [44]. Transmission of CoNS isolates from hospital settings may increase the risk of colonization and disease in patients. As a result, HPs are at risk for secondary infections caused by CoNS such as bacteremia associated with vascular catheters, cellulitis, and skin ulcers.

## 5. Conclusion

In conclusion, the high prevalence rate of antibiotic resistance and biofilm production ability was observed in *S. epidermidis* and *S. haemolyticus* isolated from HPs and HCWs. Our findings suggested that HCWs can be considered as a reservoir or methicillin-resistant CoNS for HPs. Distinguishable PFGE types among MRSE and MRSH showed the absence of the same clonality. The high rate of resistance to various antibiotics as well as the possibility of cross infection shows the importance of a pattern shift in performing the management and control programs of CoNS, especially in healthcare centers.

## Figures and Tables

**Table 1:** Characteristics of clinical specimens and frequency of *S. epidermidis* and *S. haemolyticus* in different wards and selected hospitals.

Hospitals	City	*S. epidermidis* (no)		*S. haemolyticus* (no)		Sources (no)	Wards No (%)		
		HPs	HCWs	HPs	HCWs		Infectious (%)	ICU (%)	Dialysis (%)
Pediatrics	Tabriz	15	1	1	3	B (10), TT (6), S (4)	5 (6.2)	15 (18.7)	0 (0)
Imam Reza	Tabriz	13	9	3	3	B (13), TT (1), P (2), S (12)	6 (7.4)	19 (23.5)	3 (3.7)
Sina	Tabriz	7	2	5	1	B (4), U (5), P (3), S (3)	3 (3.7)	12 (14.8)	0 (0)
Imam Hossein	Shahroud	8	7	3	0	B (9), TT (2), S (7)	5 (6.2)	9 (11.1)	4 (4.9)
Total		43	19	12	7	B (36), TT (9), U (5), P (5), S (26)	19 (23.5)	55 (68.7)	7 (8.6)

HPs: hospitalized patients; HCWs: healthcare workers; ICU: intensive care unit; B: blood; U: urine; TT: tracheal tube; P: plural fluid; S: skin.

**Table 2:** The antibiotic resistance profile of *S. epidermidis* and *S. haemolyticus* isolated from HPs and HCWs.

Antibiotics	HPs & HCWs		*P* value	HPs & HCWs		*P* value
	*S. epidermidis* (*n* = 62)	*S. haemolyticus* (*n* = 19)		HPs	HCWs	
Cefoxitin	50 (80.6)	17 (89.5)	0.37	46 (83.6)	21 (80.8)	0.75
Ciprofloxacin	28 (45.2)	15 (78.9)	0.01^∗^	31 (56.4)	12 (46.2)	0.39
Gentamicin	43 (69.4)	9 (47.4)	0.80	37 (67.3)	15 (57.7)	0.40
Chloramphenicol	19 (30.6)	9 (47.4)	0.18	25 (45.5)	3 (11.5)	0.03^∗^
Erythromycin	34 (54.8)	11 (57.9)	0.81	36 (65.5)	9 (34.6)	0.09
Clindamycin	28 (45.2)	12 (63.2)	0.17	32 (58.2)	8 (30.8)	0.02^∗^
Trimethoprim-sulfamethoxazole	52 (83.9)	14 (73.7)	0.31	52 (94.5)	14 (53.8)	<0.001^∗^
Linezolid	0	0	—	0	0	—
MDR	45 (72.6)	15 (78.9)	0.58	44 (80)	16 (61.5)	0.07

SXT: trimethoprim-sulfamethoxazole; MDR: multidrug resistance. ^∗^Statistically significant.

**Table 3:** Biofilm formation, distribution of SCC*mec* types, and the prevalence of *icaA* and *icaD* genes.

Bacteria	SCC*mec* types *N* (%)							*Ica* operon *N* (%)		Biofilm formation *N* (%)	
	I	II	III	IV	I + III	III + V	NT	*icaA*	*icaD*	Positive *N* (%)	Negative *N* (%)
HPs	14 (21.1)	2 (3)	2 (3)	7 (10.6)	7 (10.6)	2 (3)	11 (16.7)	28 (62.2)	28 (62.2)	49 (89.1)	6 (10.9)
HCWs	9 (13.6)	2 (3)	0	6 (9.1)	2 (3)	0	2 (3)	12 (57.1)	12 (57.1)	21 (80.8)	5 (19.2)
MRSE	17 (25.8)	1 (1.5)	2 (3)	12 (18.2)	8 (12.1)	2 (3)	10 (15.2)	29 (55.8)	29 (55.8)	46 (88.5)	6 (11.5)
MRSH	6 (9.1)	3 (4.5)	0	1 (1.5)	1 (1.5)	0	3 (4.5)	11 (78.6)	11 (78.6)	12 (85.7)	2 (14.3)
Total	23 (34.8)	4 (6.1)	2 (3)	13 (19.7)	9 (13.6)	2 (3)	13 (19.7)	40 (60.6)	40 (60.6)	70 (86.4)	11 (13.6)

HCWs: healthcare worker; HP: hospitalized patients; MRSE: methicillin-resistant *S. epidermidis*; MRSH: methicillin-resistant *S. haemolyticus*; NT: not typeable.

## Data Availability

All relevant data are within the manuscript.
